# Elderly Woman with Abdominal Pain: Bedside Ultrasound Diagnosis of Diverticulitis

**DOI:** 10.5811/westjem.2015.7.27549

**Published:** 2015-10-20

**Authors:** Jason D. Heiner

**Affiliations:** University of Washington, Division of Emergency Medicine, Seattle, Washington, Peace Health Peace Island Medical Center, Department of Emergency Medicine, Friday Harbor, Washington

A 72-year-old otherwise healthy female presented to the emergency department with two weeks of worsening abdominal pain. She was afebrile with normal vital signs. Her physical examination was notable for moderate abdominal tenderness without rebound to the left and suprapubic regions of the abdomen. Laboratory studies were remarkable for a white blood cell count of 13,000/mm^3^. A focused bedside ultrasound over the patient’s region of maximal discomfort revealed a thickened bowel wall and several small contiguous hypoechoic projections surrounding a hyperechoic center, suggestive of diverticulitis (Figure). She was given metronidazole and ciprofloxacin and her diagnosis of uncomplicated colonic diverticulitis was confirmed by computed tomography (CT) (Figure).

Acute diverticulitis resulting from inflammation of colonic diverticulum affects over half the population greater 65 years of age.^1^,^2^ While an estimated 85% of cases resolve with nonoperative care, complications such as large abscesses, fistula formation, perforation, and peritonitis do occur.^1^ CT is typically employed to diagnose presumed diverticulitis and recognize the presence of complicated disease, but for several decades ultrasound has been increasingly described as a similarly useful imaging modality.^1^–^5^

Particularly in cases of suspected uncomplicated diverticulitis, abdominal ultrasound may reach the diagnostic reliability of CT.^1^,^2^,^4^,^5^ Ultrasound may detect edema leading to loss of normal bowel architecture, identify inflamed diverticula, and expose mesenteric or omental fat.^2^–^4^ Key described songraphic findings include the following: edematous diverticula with thickened hypoechoic walls and hyperechoic centers, air containing diverticula with subsequent hyperechoic acoustic shadowing artifact, enlarged colonic walls greater then 5mm, and surrounding hyperechoic zones representing inflamed fat.^1^–^4^ Focused bedside imaging of the area of pain and tenderness may aid in initiation of early antibiotic treatment pending any additional confirmatory studies, but imaging can be hindered by neighboring bowel gas and CT or other complementary imaging may be warranted to search for complications or reveal alternative diagnoses.^1^,^2^

## Figures and Tables

**Figure:**
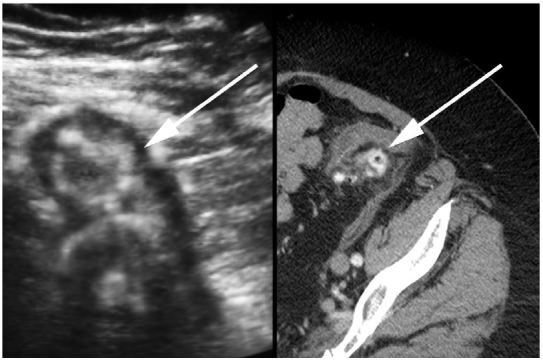
Transabdominal sonographic view of the patient’s abdomen (Left) and associated axial computed tomography (Right) revealing inflamed diverticula (arrow). Related imaging findings of diverticulitis such as edematous diverticula with thickened hypoechoic walls and hyperechoic centers, and surrounding hyperechoic zones representing inflamed fat.
